# Supplementary material for: Prostacyclin Analogues Inhibit Platelet Reactivity, Extracellular Vesicle Release and Thrombus Formation in Patients with Pulmonary Arterial Hypertension
Source: J Clin Med. 2021 Mar 2;10(5):1024. doi: 10.3390/jcm10051024 (PMC7958838; doi:10.3390/jcm10051024)
Supplement: Supplementary file 1 [file jcm-10-01024-s001.zip › jcm-1093425 SI/jcm-1093425 Supplementary.pdf]

MIFlowCyt-EV of clinical research study “Prostacyclin analogues inhibit platelet reactivity, extracellular vesicle release and thrombus formation in patients with pulmonary arterial hypertension”

1 Flow cytometry

1.1 Experimental design

The aim of flow cytometry (A60-Micro, Apogee Flow Systems, Hemel Hempstead, UK) was to compare the concentrations of extracellular vesicles (EVs) released from platelets ($CD61^+$), activated platelets ($CD61^+/CD62P^+$), leukocytes ($CD45^+$) and endothelial cells ($CD146^+$) in platelet-depleted plasma (PDP) of patients with pulmonary arterial hypertension (PAH) treated with prostacyclin analogues and PAH patients not receiving prostacyclin analogues. We hypothesised that (1) patients with PAH treated with prostacyclin analogues have lower platelet reactivity and a lower EV concentration compared to patients not receiving prostacyclin analogues, and (ii) the antiplatelet effects as well as EV concentrations in patients treated with prostacyclin analogues (apoprostenol, treprostinil and iloprost) may vary. Pre-analytical variables can be found in the manuscript.

All samples were measured using an autosampler, which facilitates subsequent measurements of samples in a 96-well plate. The entire study involved six 96-well plates that were measured within 4 subsequent days. Each well plate contained buffer-only controls, unstained controls, antibody in buffer controls, and isotype controls. Flow rate and scatter calibrations were performed daily. Fluorescence calibration was performed once. To automatically determine optimal samples dilutions, apply calibrations, determine and apply gates, generate reports with scatter plots and generate data summaries, we developed and applied custom-build software (MATLAB R2018b, Mathworks, Natick, MA, USA).

1.2 Sample dilutions

Because the particle concentration in PDP differs between individuals, samples require different dilutions to avoid swarm detection [2] and to achieve statistically significant counts within a clinically applicable measurement time. Although serial dilutions are recommended

to find the optimal dilution, we consider serial dilutions unfeasible in a study with 40 donors at a single timepoint. Therefore, we developed a procedure to estimate to optimal sample dilution (Section 1.2 of https://doi.org/10.6084/m9.figshare.c.4753676). In sum, we showed that for our flow cytometer and settings used, a count rate ≤ $5.0 \cdot 10^3$ events second unlikely results in swarm detection.

To find the dilution resulting in a count rate ≤$5.0 \cdot 10^3$ events per second, we diluted each PDP sample 200 and 2,000-fold in citrated (032%) phosphate buffered saline (PBS) and measured the total concentration of particles for 30 seconds without staining. For all experiments, citrated (0.32%) PBS was filtered (Whatman 50 nm, Sigma-Aldrich, US) and had a pH of 7.4. By diluting each sample 200-fold and 2,000-fold, all but 2 samples had for at least one of the two measurements a count rate ≥321 (statistically significant) and ≤$5.0 \cdot 10^3$ (no swarm detection) events per second. Figure S1A shows a distribution of the measured total particle concentrations of all samples in the study. Taking into account the measured concentration and flow rate, we calculated the minimum pre-dilution required before staining (next section) to achieve a count rate ≤$5.0 \cdot 10^3$ events per second after staining. The staining procedure adds an extra dilution of 11.3-fold to the overall dilution. To simplify procedures, samples were divided into 13 categories of pre-dilution: 2-fold, 5-fold, 10-fold, 20-fold, 30-fold, 40-fold, 65-fold, 100-fold, 130-fold, 150-fold, 250-fold, 300-fold, and 1,500-fold. Figure S1B shows a distribution of the applied pre-dilutions of all samples in the study.

1.3 EV staining

EVs in PDP were stained with antibodies and lactadherin. Prior to staining, antibodies were diluted in citrated (0.32%) PBS and centrifuged at 18,890 g for 5 min to remove aggregates. Table S1 shows an overview of the used reagents and antibody concentrations during staining. Each sample was double labelled with CD45-APC (allophycocyanin) and CD146-PE (phycoerythrin), and with CD61-APC and CD62p-PE. To stain, 20 μL of pre-diluted (Figure S1B) PDP was incubated with 2·2.5 μL of antibodies or isotype controls and kept in the dark for 2 h at room temperature. Post-staining, samples were diluted 11.3-fold in 200 μL of citrated (0.32%) PBS to decrease background fluorescence from unbound reagents.

1.4 Buffer-only control

Each 96-wellplate contained at least 1 well with filtered (Whatman 50 nm) PBS, which was measured with the same flow cytometer and acquisition settings as all other samples. The

mean count rate was 54.9 events per second, which is substantially lower than the target count rate ($2.5\text{-}5.0 \cdot 10^3$ events per second) for PDP samples.

1.5 Buffer with reagents control

Each 96-wellplate contained a buffer with reagent control for each reagent (Table S1), which was measured with the same flow cytometer and acquisition settings as all other samples. For all reagents, the count rate of the buffer with reagents control was lower than the count rate of the buffer-only control.

1.6 Unstained controls

Unstained controls were measured at the same dilution and settings as the stained samples. Unstained controls were not used during data analysis.

1.7 Isotype controls

Table S1 shows an overview of the used isotype controls. For all isotype controls, the count rate of the buffer with isotype control antibodies was lower than the count rate of the buffer-only control. For particles with a diameter >200 nm and a refractive index <1.42, as reported in this study, we measured on average 4.6 APC+ events for IgG-APC and 10.0 PE+ events for IgG-PE during 120 seconds.

1.8 Trigger channel and threshold

Based on the buffer-only control (54.9 events s^{-1}), the acquisition software was set up to trigger at 14 arbitrary units SSC, which is equivalent to a side scattering cross section of 10 nm^2 (Rosetta Calibration, v1.16, Exometry, Amsterdam, The Netherlands).

1.9 Flow rate quantification

Each measurement day, we used 110 nm FITC beads with a specified concentration (Apogee calibration beads, Apogee Flow Systems, Hemel Hempstead, UK) to calibrate the flow rate of the A60-Micro. Fig. S2 shows the measured flow rate per measurement day. The adjusted flow rate is 3.01 µL/min and the measured median flow rate is 2.92 ± 0.23 uL/min (mean ± standard deviation). Because the A60-Micro is equipped with a syringe pump with volumetric control, we assumed a flow rate of 3.01 µL/min for all measurements.

1.10 Fluorescence calibration

Calibration of the fluorescence detectors from arbitrary units (a.u.) to molecules of equivalent soluble fluorochrome (MESF) was accomplished using 2 µm Q-APC beads (2321-175, BD)

and SPHERO Easy Calibration Fluorescent Particles (ECFP-F2-5K, Spherotech Inc., Irma Lee Circle, IL, USA).

The 2 µm Q-APC beads did not result in linear relation between the measured mean fluorescence intensity (MFI) and the specified MESF, probably because scattered light is leaking to the APC detector. Therefore, we applied a correction as explained in https://www.doi.org/10.6084/m9.figshare.11340386. For each measurement, we added fluorescent intensities in MESF to the flow cytometry data files by custom-build software (MATLAB R2018a) using following equation:

$\mathrm{I(MESF)} = 10^{a \cdot \log_{10} \mathrm{I(a.u.)} + b}$	Equation S1

where I is the fluorescence intensity, and *a* and *b* are the slope and the intercept of the linear fits in figures S3A-B, respectively.

1.11 Light scatter calibration

We used Rosetta Calibration to relate scatter measured by FSC or SSC to the scattering cross section and diameter of EVs. Figure S4 shows print screens of the scatter calibrations. We modelled EVs as core-shell particles with a core refractive index of 1.38, a shell refractive index of 1.48, and a shell thickness of 6 nm. For each measurement, we added the FSC and SSC scattering cross sections and EV diameters to the flow cytometry datafiles by custom-build software (MATLAB R2018a). The SSC trigger threshold corresponds to a side scattering cross section of 10 nm^2.

1.12 EV diameter and refractive index approximation

Flow-SR was applied to determine the size and refractive index of particles and improve specificity by enabling label-free differentiation between EVs and lipoprotein particles [3,4]. Flow-SR was performed as previously described [3,4]. Lookup tables were calculated for diameters ranging from 10 to 1000 nm, with step sizes of 1 nm, and refractive indices from 1.35 to 1.80 with step sizes of 0.001. The diameter and refractive index of each particle was added to the .fcs file by custom-build software (MATLAB R2018a).

Because Flow-SR requires accurate measurements of both FSC and SSC, we applied Flow-SR only to particles with diameters >200 nm and fulfilling the condition:

$\mathrm{SSC(nm^2)} > -0.7 \cdot \mathrm{FSC(nm^2)} + 2.5$	Equation S2

1.13 MIFlowCyt checklist

The MIFlowCyt checklist is added to Table S2.

1.14 EV number concentration

The concentrations reported in the manuscript describe the number of particles (1) that exceeded the SSC threshold, corresponding to a side scattering cross section of 10 nm^2, (2) that were collected during time intervals, for which the count rate was within 50% of the median count rate, (3) with a diameter >200 nm as determined by Flow-SR [3], (4) fulfilling the condition of equation S2, (5) having a refractive index <1.42 to omit false positively labeled chylomicrons, and (6) are positive at the corresponding fluorescence detector(s), per mL of PDP.

For the samples stained with CD61-APC and CD62-PE, two extra gates were applied between aforementioned steps 2 and 3. To omit residual platelets, only events with a side scattering cross section <2,000 nm^2 and an APC intensity <7,000 MESF were included. To omit CD61 aggregates, only events fulfilling the condition:

$\mathrm{SSC(nm^2)} > 0.9 \cdot \mathrm{APC(MESF)} - 1.2$	Equation S3

were included. For the samples stained with CD45-APC and CD146-PE, one extra gate was applied between aforementioned steps 2 and 3. To omit CD45 aggregates, only events fulfilling the condition:

$\mathrm{SSC(nm^2)} > 0.8 \cdot \mathrm{APC(MESF)} - 1.5$	Equation S4

were included.

1.15 Data sharing

Raw data, data with standard units, and a summary of all flow cytometry scatter plots and gates applied are available via https://www.doi.org/10.21942/uva.13067603.

2 References

[1] Sluyter R. P2X and P2Y receptor signaling in red blood cells. *Front Mol Biosci* Frontiers; 2015; **2**: 60.

[2] van der Pol E, van Gemert MJC, Sturk A, Nieuwland R, van Leeuwen TG. Single vs. swarm detection of microparticles and exosomes by flow cytometry. *J Thromb Haemost* 2012; **10**: 919–30.

[3] van der Pol E, de Rond L, Coumans FAW, Gool EL, Böing AN, Sturk A, Nieuwland R, van Leeuwen TG. Absolute sizing and label-free identification of extracellular vesicles by flow cytometry. *Nanomedicine Nanotechnology, Biol Med* 2018; **14**: 801–10.

[4] de Rond L, Libregts SFWM, Rikkert LG, Hau CM, van der Pol E, Nieuwland R, van Leeuwen TG, Coumans FAW. Refractive index to evaluate staining specificity of extracellular vesicles by flow cytometry. *J Extracell vesicles* 2019; .

Figures

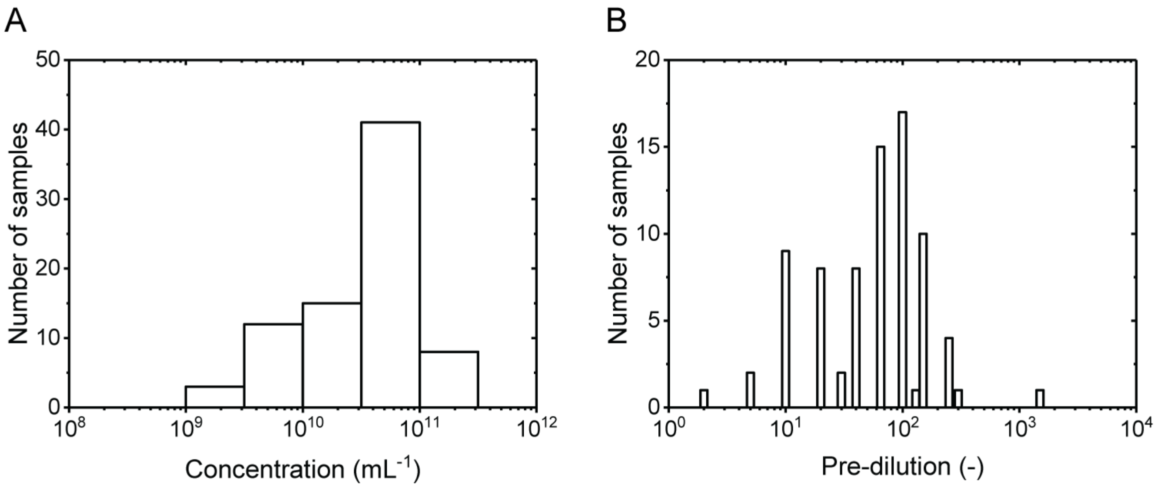

Figure S1. **Distributions of (A) measured concentration of particles exceeding the side scatter threshold and (B) applied pre-dilutions of all samples in the study.** The side scatter threshold corresponds to a scattering cross section of ~10 nm^2. The measured particle concentrations differ 3 orders of magnitude, requiring pre-dilutions ranging from 2-fold to 1,500-fold.

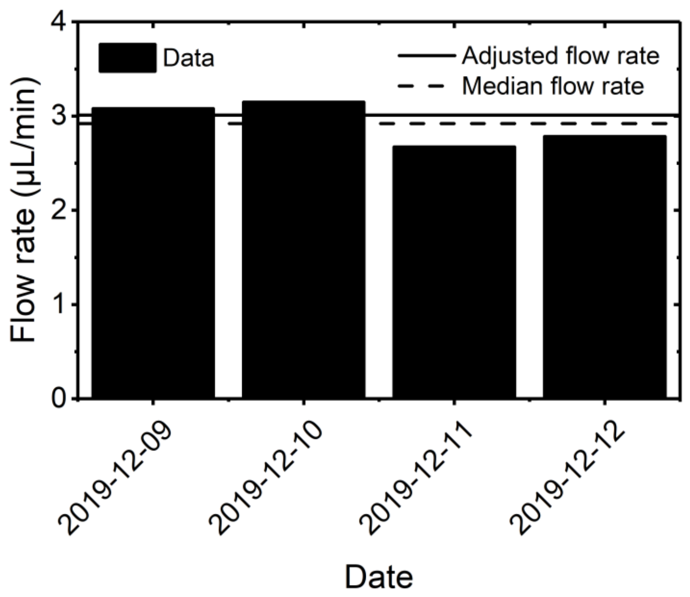

Figure S2. **Measured flow rate at the A60-Micro versus date that experiments were performed.** The adjusted flow rate is 3.01 µL/min and the measured median flow rate is 2.92 µL/min. For all days the measured flow rate was within 11% of the adjusted flow rate.

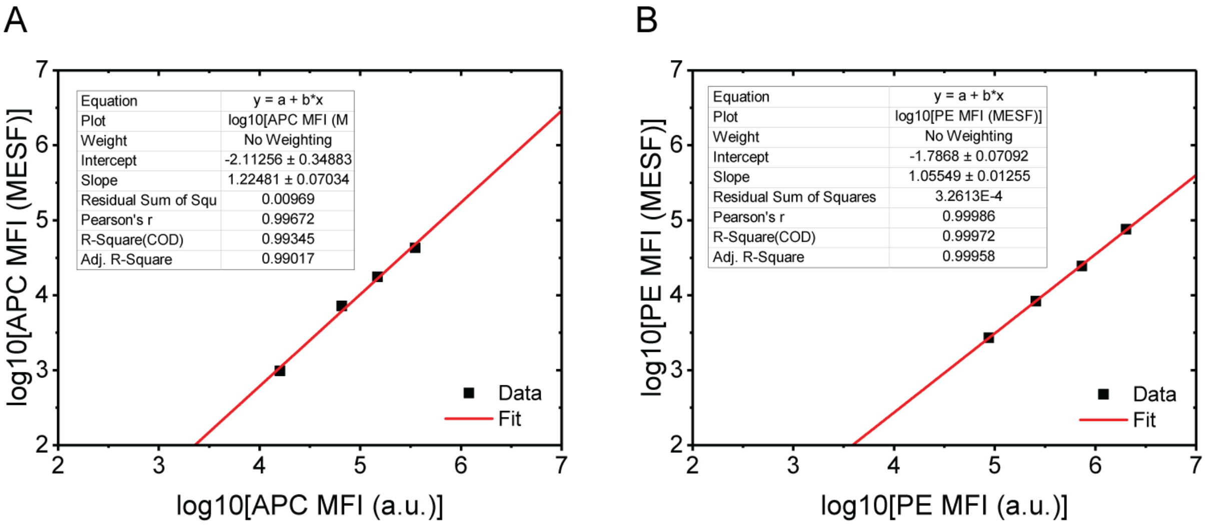

Figure S3. Calibration of the fluorescence detectors from arbitrary units (a.u.) to molecules of equivalent soluble fluorochrome (MESF). Logarithmic MESF versus logarithmic mean fluorescence intensity (MFI) for (A) APC and (B) phycoerythrin (PE). Data (symbols) are fitted with a linear function (line), resulting in a slope of 1.22 and intercept of -2.11 for APC, a slope of 1.05 and intercept of -1.79 for PE.

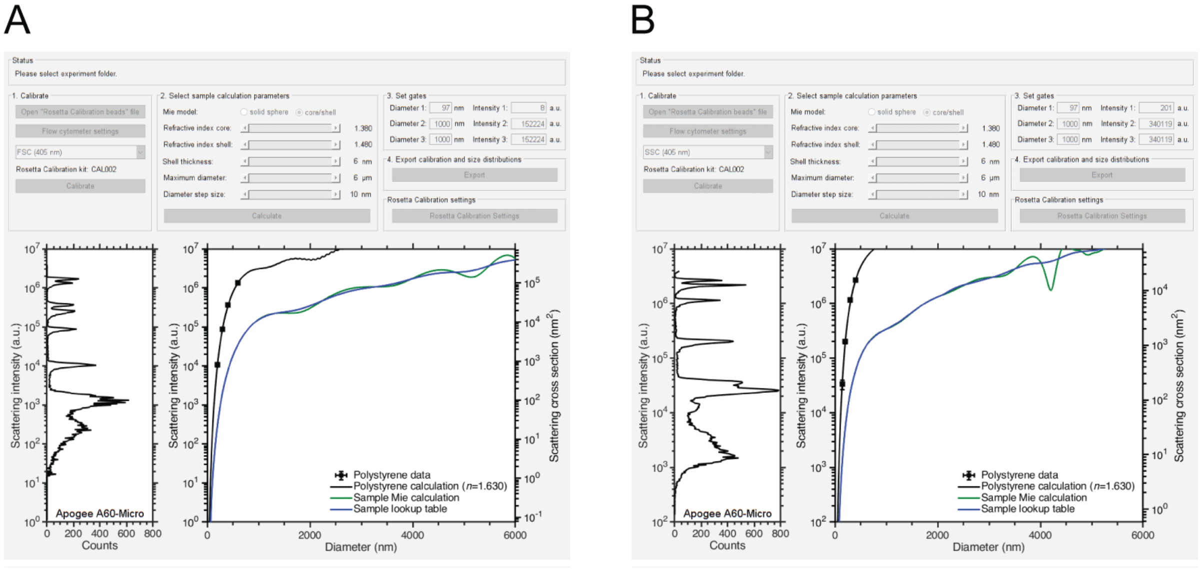

Figure S4. Forward scatter and side scatter calibration of the A60-Micro by Rosetta Calibration. To relate scatter to the diameter of EVs, we modelled EVs as core-shell particles with a core refractive index of 1.38, a shell refractive index of 1.48, and a shell thickness of 6 nm.

Table S1: Overview of staining reagents. Characteristics being measured, analyte, analyte detector, reporter, isotype, clone, concentration, manufacturer, catalog number and lot number of used staining reagents. The antibody concentration during measurements was 11.3-fold lower than the antibody concentration during staining.

**Characteristic measured**	**Analyte**	**Analyte detector**	**Reporter**	**Isotype**	**Clone**	**Concentration during staining (μg mL^{-1})**	**Manufacturer**	**Catalog number**	**Lot number**
Integrin	Human CD61	Anti-human CD61 antibody	APC	IgG1	Y2/51	50	Dako	C7280	200483345
Adhesion molecule	Human CD62p	Anti-human CD62P antibody	PE	IgG1	CLB Thromb/6	6.25	Beckman Coulter	IM1759U	37
Leukocyte common antigen	CD45	Anti-human CD45 antibody	APC	IgG1	2D1	25	Beckman Dickinson	340910	5040555
Adhesion molecule	CD146	Anti-human CD146 antibody	PE	IgG1	S-Endo 1	3.75	Biocytex	5050-PE100T	173455
Affinity for Fc receptor	Fc receptor	IgG1	APC	n.a.	X40	200	Beckman Dickinson	554681	7075605
	Fc receptor	IgG1	PE	n.a.	IS5-21F5	50	Beckman Dickinson	345816	7248665

APC: allophycocyanin; CD: cluster of differentiation; IgG: immunoglobulin G; PE: phycoerythrin.

Table S2. MIFlowCyt checklist.

**Requirement**	**Please Include Requested Information**
1.1. Purpose	To compare the concentrations of extracellular vesicles (EVs) released from platelets ($CD61^{+}$), activated platelets ($CD61^{+}$/$CD62P^{+}$), leukocytes ($CD45^{+}$) and endothelial cells ($CD146^{+}$) in platelet-depleted plasma (PDP) of patients with pulmonary arterial hypertension (PAH) treated with prostacyclin analogues and PAH patients not receiving prostacyclin analogues.
1.2. Keywords	Prostacyclin analogues, pulmonary arterial hypertension, platelet reactivity, extracellular vesicles, thrombus formation
1.3. Experiment variables	Treatment with or without prostacyclin analogues. Treatment with different prostacyclin analogues (apoprostenol, treprostinil and iloprost).
1.4. Organization name and address	Amsterdam University Medical Centers<br>Location Academic Medical Centre<br>Meibergdreef 9<br>1105 AZ Amsterdam<br>The Netherlands
1.5. Primary contact name and email address	Aleksandra Gąsecka, a.gasecka@amc.uva.nl
1.6. Date or time period of experiment	December 2019
1.7. Conclusions	PAH patients treated with prostacyclin analogues had comparable concentrations of EVs from all platelets and megakaryocytes (CD61+), but lower concentrations of EVs from activated platelets (CD62P+) and leukocytes, compared to control patients ($p=0.04$ and, $p=0.01$, respectively). There was also a trend towards a lower concentration of EVs from endothelial cells ($p=0.08$). Whereas treprostinil had no significant effect on any subtype of EVs, epoprostenol decreased the concentrations of platelet EVs (CD61+ and CD62P+, $p=0.04$ for both) and leukocyte EVs ($p=0.02$).
1.8. Quality control measures	All samples were measured using an autosampler, which facilitates subsequent measurements of samples in a 96-well plate

	(see MIFlowCyt 1.9). Each well plate contained buffer-only controls (section S1.4), antibody in buffer controls (section S1.5), unstained controls (section S1.6) and isotype controls (section S1.7). The flow rate was calibrated with Apogee calibration beads (Apogee Flow Systems, Hemel Hempstead, UK). Fluorescence detectors were calibrated (section S1.10) with 2 µm Q-APC beads (2321-175, BD) and SPHERO Easy Calibration Fluorescent Particles (ECFP-F2-5K, Spherotech Inc., Irma Lee Circle, IL, USA). FSC and SSC were calibrated with Rosetta Calibration (v1.16, section S1.11).
1.9 Other relevant experiment information	The entire study involved six 96-well plates that were measured during 4 subsequent days.
2.1.1.1. Sample description	Thawed PDP (MIFlowCyt 2.1.1.2) from hospitalized humans with PAH (MIFlowCyt 2.1.1.3).
2.1.1.2. Biological sample source description	Blood was collected in two 1.6 mL hirudin plastic tubes and one 8.2 mL 0.109 M citrated (S-Monovette, Sarstedt) via antecubital vein puncture using a 19-gauge needle, without tourniquet. The first 2 mL were discarded to avoid pre-activation of platelets. Platelet reactivity and platelet-rich thrombus formation were evaluated simultaneously by two operators using impedance aggregometry and whole blood perfusion system, respectively, available at the 1st Chair and Department of Cardiology, Medical University of Warsaw, Poland. Both measurements were done and within 5 minutes after blood collection due to the short half-life time of prostacyclin analogues (epoprostenol: 6 minutes, iloprost: 20-30 minutes, treprostinil: 1.5 hour). Platelet-depleted plasma for EV analysis was prepared using double centrifugation with a Rotina 380 R, equipped with a swing-out rotor and a radius of 155 mm (Hettich Zentrifugen, Tuttlingen, Germany). The centrifugation parameters were: 2,500 g, 15 minutes, 20˚C, acceleration speed 1, no brake. The first centrifugation step was done with 8.2 mL citrate-anticoagulated whole blood collection tubes. Supernatant was collected 10 mm above the buffy coat. The

	second centrifugation step was done with 3.5 mL plasma in 15 mL polypropylene centrifuge tubes (Greiner Bio-One B.V). Supernatant (platelet-depleted plasma) was collected 5 mm above the buffy coat, transferred into 5 mL polypropylene centrifuge tubes (Greiner Bio-One B.V), mixed by pipetting, transferred to 1.5 mL low-protein binding Eppendorfs (Thermo Fisher Scientific) and stored in -80˚C until analysed. Prior to analysis, samples were thawed for 1 minute in a water bath (37 °C) to avoid cryoprecipitation.
2.1.1.3. Biological sample source organism description	Hospitalized humans with PAH (for inclusion criteria, please see Table 1 of the manuscript).
2.2 Sample characteristics	PDP is expected to contain EVs, lipoproteins and proteins.
2.3. Sample treatment description	Please see section S1.3.
2.4. Fluorescence reagent(s) description	Please see Table S1.
3.1. Instrument manufacturer	Apogee, Hemel Hempstead, UK
3.2. Instrument model	A60-Micro
3.3. Instrument configuration and settings	Samples were analysed for 2 minutes at a flow rate of 3.01 µL/min on an A60-Micro, equipped with a 405 nm laser (100 mW), 488 nm laser (100 mW) and 638 nm laser (75 mW). The trigger threshold was set at SSC 14 arbitrary units, corresponding to a side scattering cross section of 10 nm^2 (Rosetta Calibration). For FSC and SSC, the PMT voltages were 380 V and 360 V, respectively. For all detectors, the peak height was analysed. APC signals were collected with the 638-D Red(Peak) detector (long pass 652 nm filter, PMT voltage 510 V). PE signals were collected with the 488-Orange(Peak) detector (575/30 nm band pass filter, PMT voltage 520 V).
4.1. List-mode data files	Raw data, data with standard units and a summary of all flow cytometry scatter plots and gates applied are available via https://www.doi.org/10.21942/uva.13067603.

4.2. Compensation description	No compensation was required because no fluorophore combinations were used that have overlapping emission spectra.
4.3. Data transformation details	No data transforms were applied.
4.4.1. Gate description	To automatically apply gates, generate pdf reports with scatter plots, and summarize the data in a table, custom-build software (MATLAB R2018b) was used. Please find below a description of the gates.<br>First, only events that were collected during time intervals, for which the count rate was within 50% of the median count rate, were included.<br>Second, for the samples stained with CD61-APC and CD62-PE, two gates were applied. To omit residual platelets, only events with a side scattering cross section <2,000 and an APC intensity <7,000 MESF were included. To omit CD61 aggregates, only events fulfilling equation S3 were included. For the samples stained with CD45-APC and CD146-PE, one extra gate was applied between aforementioned steps 2 and 3. To omit CD45 aggregates, only events fulfilling the equation S4 were included.<br>Third, to include particles within the dynamic range of Flow-SR [3], particles with a diameter >200 nm and fulfilling the condition of equation S2 were included.<br>Fourth, to exclude false positively labeled chylomicrons and thus primarily include EVs, only particles with a refractive index <1.42 were included.<br>Fifth, fluorescence gates were automatically determined with a mathematical algorithm (MATLAB R2018b) and applied. Lower bounds of the fluorescent gates are 160 MESF for CD61-APC, 109 MESF for CD62p-PE, 140 MESF for CD45-APC, and 88 MESF for CD146-PE.
4.4.2. Gate statistics	The number of positive events was corrected for flow rate, measurement time and dilutions performed during sample preparation.

4.4.3. Gate boundaries	On overview of all gates can be found in the compressed data summary files (https://www.doi.org/10.21942/uva.13067603)

CD: cluster of differentiation; EVs: extracellular vesicles; FSC: forward scattering; PDP: platelet free plasma; SSC: side scattering.

Supplementary Figures for the manuscript "Prostacyclin analogues inhibit platelet reactivity, extracellular vesicle release and thrombus formation in patients with pulmonary arterial hypertension"

Figure S5. Correlations between the dose of treprostinil and platelet function parameters. AUC: area under curve. Values analysed using Spearman correlation coefficient.

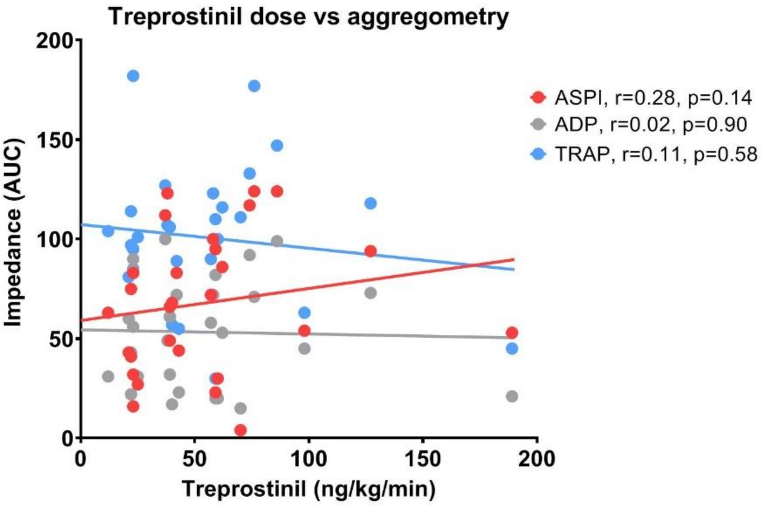

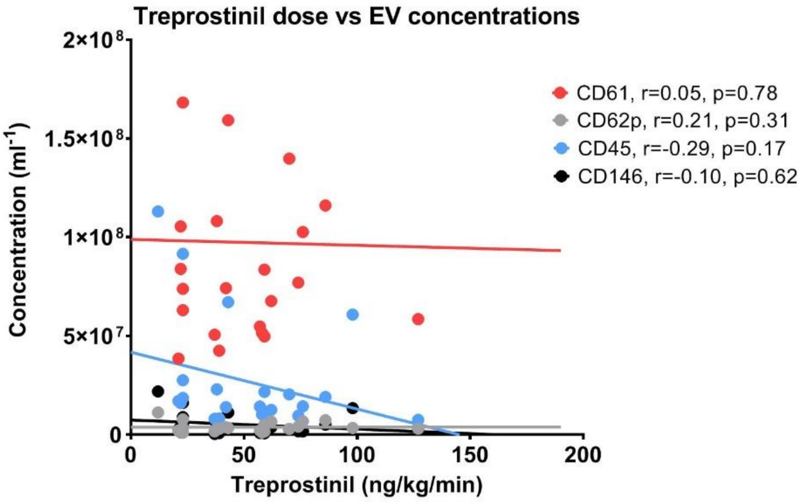

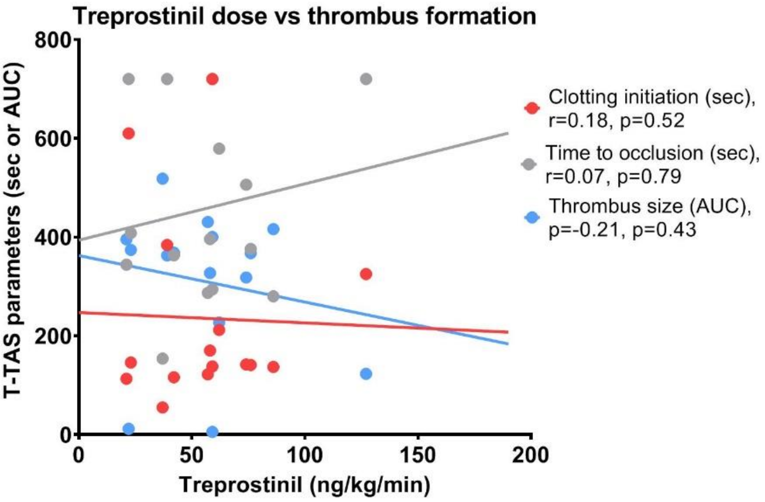

Figure S6. Comparison of platelet function parameters between female and male patients. AUC: area under curve. Values compared using Mann–Whitney U test and showed as median and interquartile range.

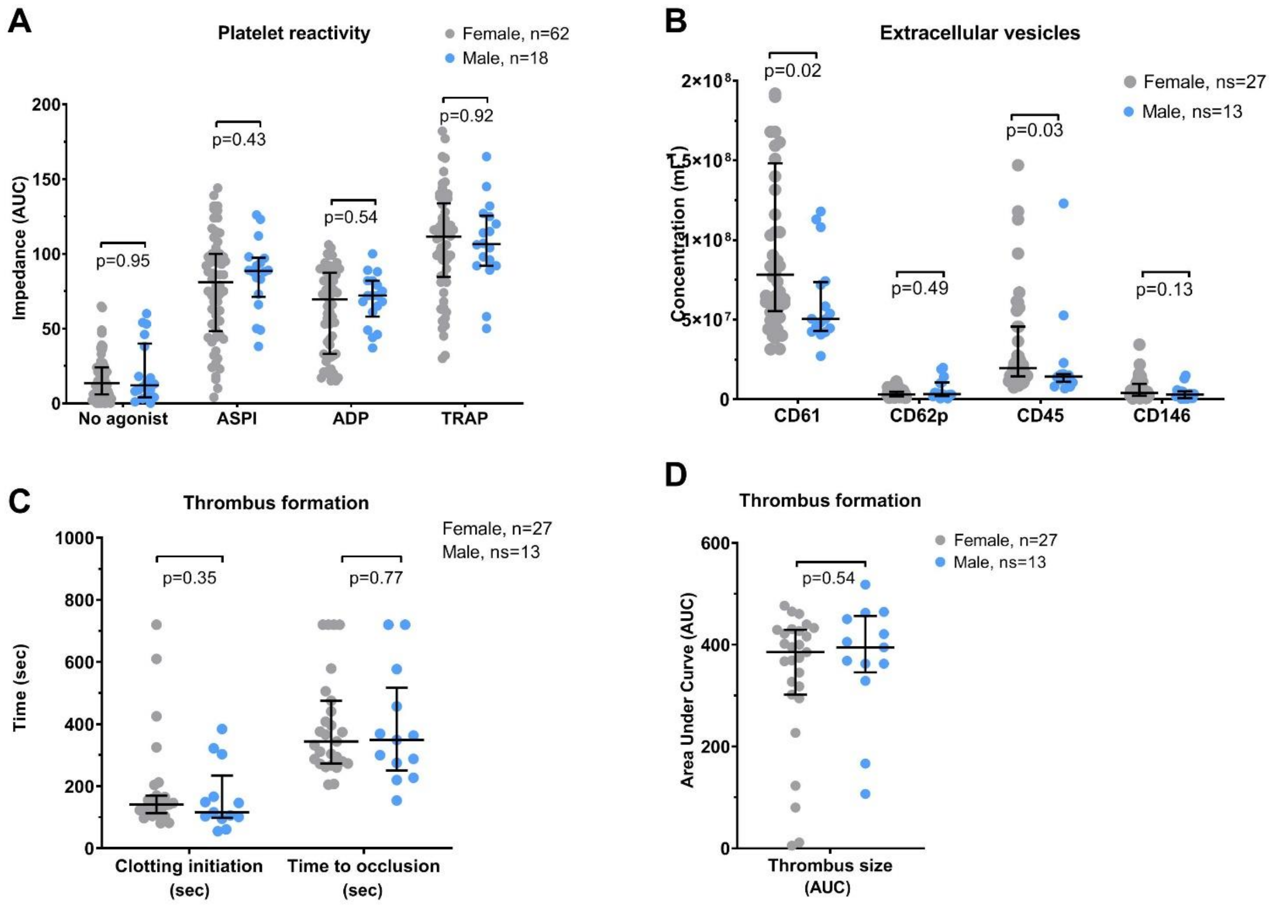

Table S3. Multivariate logistic regression including the association between the concentrations of $CD61^{+}$ EVs above the median value and clinical variables (age, gender, WHO class and treatment with PGI^{2} analogues).

Variable	OR	95% CI		p-value
		Lower	Upper	
Age	1.026	0.984	1.070	0.228
**Gender (female)**	**6.672**	**1.373**	**32.425**	**0.019**
WHO class	1.898	0.655	5.502	0.238
**Treatment with PGI^{2} analogues**	**0.281**	**0.081**	**0.973**	**0.045**

CI: confidence interval; EF: ejection fraction, OR: odds ratio;

Table S4. Multivariate logistic regression including the association between the concentrations of $CD45^{+}$ EVs above the median value and clinical variables (age, gender, WHO class and treatment with PGI^{2} analogues).

Variable	OR	95% CI		p-value
		Lower	Upper	
Age	1.032	0.983	1.083	0.208
**Gender (female)**	**51.566**	**4.266**	**623.303**	**0.002**
WHO class	1.245	0.326	4.753	0.748
**Treatment with PGI^{2} analogues**	**4.537**	**1.169**	**17.609**	**0.029**
